# Mechanical stretch induces Ca^2+^ influx and extracellular release of PGE_2_ through Piezo1 activation in trabecular meshwork cells

**DOI:** 10.1038/s41598-021-83713-z

**Published:** 2021-02-17

**Authors:** Takatoshi Uchida, Shota Shimizu, Reiko Yamagishi, Suzumi M. Tokuoka, Yoshihiro Kita, Megumi Honjo, Makoto Aihara

**Affiliations:** 1 Department of Ophthalmology, Graduate School of Medicine, The University of Tokyo, 7-3-1, Hongo, Bunkyo-ku, Tokyo, 113-8655 Japan; 2 Senju Laboratory of Ocular Science, Senju Pharmaceutical Co., Ltd., Kobe, Japan; 3 Department of Lipidomics, Graduate School of Medicine, The University of Tokyo, Tokyo, Japan; 4 Life Science Core Facility, Graduate School of Medicine, The University of Tokyo, Tokyo, Japan

**Keywords:** Lipidomics, Cell biology, Molecular biology

## Abstract

The trabecular meshwork (TM) constitutes the main pathway for aqueous humor drainage and is exposed to complex intraocular pressure fluctuations. The mechanism of homeostasis in which TM senses changes in intraocular pressure and leads to normal levels of outflow resistance is not yet well understood. Previous reports have shown that Piezo1, a mechanically-activated cation channel, is expressed in TM and isolated TM cells. Therefore, we tested hypothesis that Piezo1 may function in response to membrane tension and stretch in TM. In human trabecular meshwork (hTM) cells, *PIEZO1* was showed to be abundantly expressed, and Piezo1 agonist Yoda1 and mechanical stretch caused a Piezo1-dependent Ca^2+^ influx and release of arachidonic acid and PGE_2_. Treatment with Yoda1 or PGE_2_ significantly inhibited hTM cell contraction. These results suggest that mechanical stretch stimuli in TM activates Piezo1 and subsequently regulates TM cell contraction by triggering Ca^2+^ influx and release of arachidonic acid and PGE_2_. Thus, Piezo1 could acts as a regulator of intraocular pressure (IOP) within the conventional outflow pathway and could be a novel therapeutic strategy to modulate IOP in glaucoma patients.

## Introduction

Intraocular pressure (IOP) is regulated by the production and outflow of aqueous humor and has been associated with increased risk of glaucoma development. The main route of aqueous humor drainage is the conventional outflow pathway, which involves the flow of aqueous humor to the Schlemm's canal through the juxtacanalicular trabecular meshwork (TM)^1,2^. Conventional outflow tissues are constantly subjected to fluctuations in IOP, and cells in these tissues sense alterations in IOP as mechanical stretch or strain^2,3^. Changes in pressure of 8–30 mmHg can cause length extension by 50% in cells in the outflow pathway^4,5^. In response to mechanical stretch or strain, TM cells activate intracellular signaling pathways leading to changes in gene expression, extracellular matrix (ECM) turnover, contractile properties^2,6^.

The non-selective cation channel TRPV4 and the two-pore domain potassium channel TREK1 are expressed in TM cells, sense mechanical stretch generated by fluctuations in IOP, and play an essential role in IOP adjustment^7–10^. Following membrane stretch, TM-resident TRPV4 channels initiate calcium (Ca^2+^) signals and cytoskeletal reorganization, and TRPV4 antagonists increased the outflow facility and lowered IOP in glaucomatous mouse eyes^10^. Recent studies showed that TREK-1 collaborates with TRPV4, regulating TM tensile homeostasis^8^. Although numerous studies highlighted the importance of mechanosensation in modulating TM outflow, the molecular links between mechanotransduction, Ca^2+^ homeostasis, and reorganization of the conventional outflow pathway remain unclear. Hence, the mechanosensory mechanisms mediating force-coupling and IOP regulation should be further explored.

Piezo1 and Piezo2 were first described in 2010 as components of a mechanically activated cation channel expressed in neurons and neuronal cell lines^11^. Piezo1 has been implicated in mechanical stretch response and extracellular Ca^2+^ influx regulation in the bladder urothelium^12^, red blood cell^13^, and neural stem cells^14^. Nevertheless, its role in TM remains poorly understood. We hypothesized that in TM, Piezo1 might respond to mechanical stress and regulate IOP by locally releasing lipid mediators. Lipid mediators are well known to have an important role in various physiological events through rapid production from the cell membrane and working transiently and locally in the restricted tissue. Notably, several arachidonic acid-derived metabolites have been implicated in mechanical signaling^15,16^. Among lipid mediators, prostaglandins such as PGF_2α_ and PGE_2_, lysophospholipids, or cannabinoids are associated to the IOP regulation^17–20^. Thus, we investigated the involvement of Piezo1 on the mechanical stress and the release of lipid mediators in primary cultured human TM cells.

## Results

### Expression of Piezo and TRPV family members in primary hTM cells

**Figure 1:**
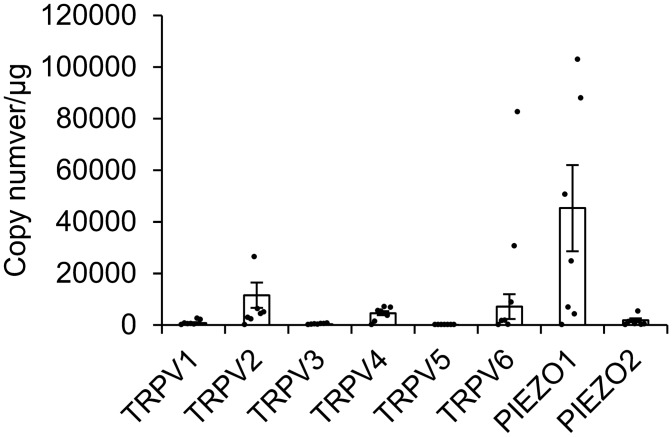
Expression levels of TRPV and Piezo family in human trabecular meshwork cells. mRNA levels of TRPV1-6 and Piezo1-2 in the human trabecular meshwork (hTM) cells were determined by qPCR. The number of mRNA copies of the target genes was calculated by normalizing to the amount of total RNA. Data are presented as means ± SD (n = Eight hTM cell populations from eight donors).

First, we examined the expression of human Piezo and TRPV family members in primary hTM cells by qPCR. Piezo1 mRNA levels were profoundly higher than Piezo2 and TRPV family mRNA levels in hTM cells (Fig. 1).

### Knockdown of Piezo1 using siRNA in hTM cells

**Figure 2:**
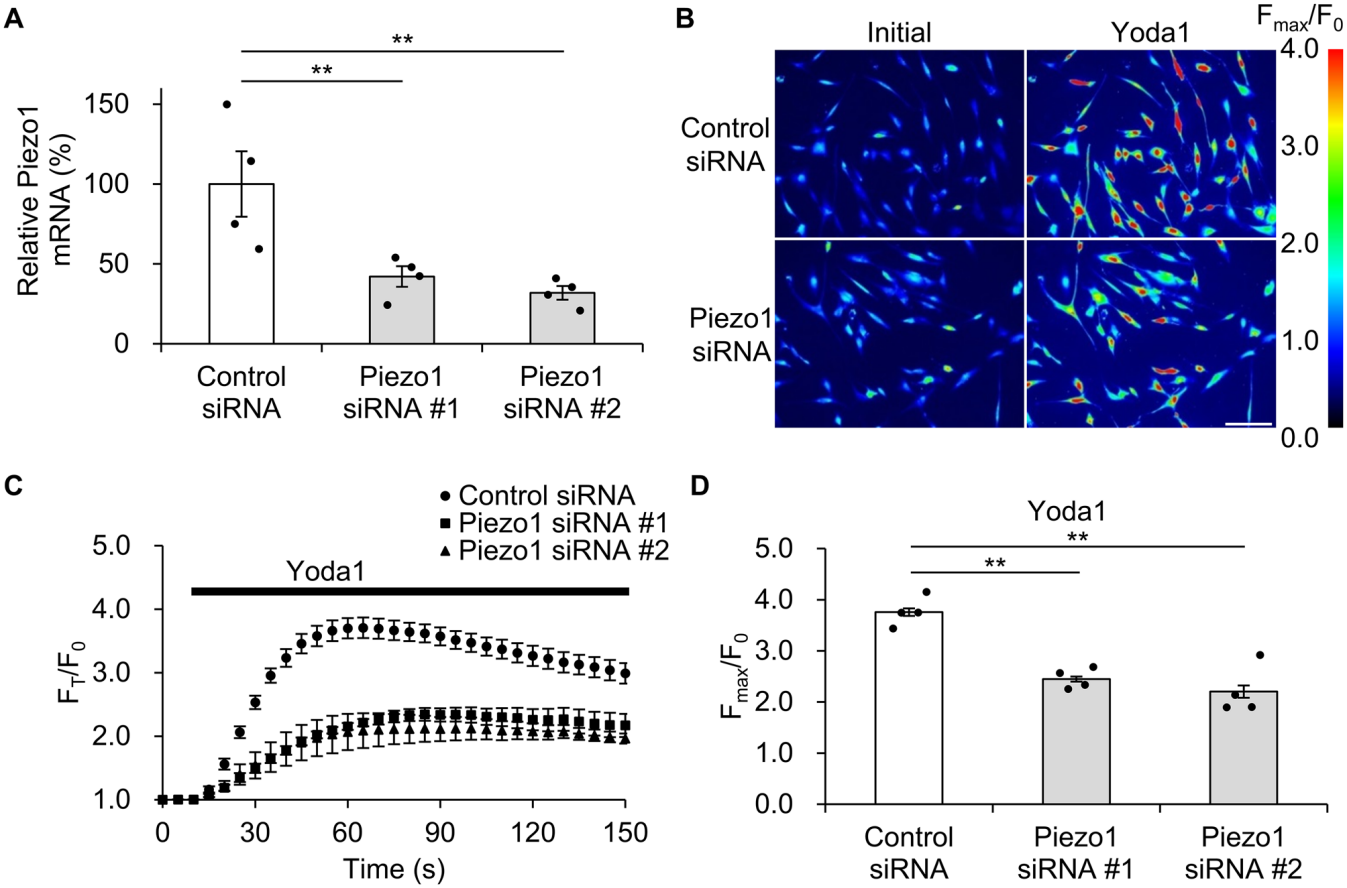
Efficacy of Piezo1 knockdown in human trabecular meshwork cells. (**A**) Relative Piezo1 mRNA levels in the human trabecular meshwork treated with control or Piezo1 siRNAs 24 h after transfection, as determined by qPCR. Data are normalized to GAPDH mRNA level. Data are presented as means ± SE (n = 4 samples of cDNA synthesized from RNA extracted from cells in 4 wells). **p < 0.01, Dunnett’s test. (**B**) Representative images illustrating intracellular Ca^2+^ changes in human trabecular meshwork cells transfected with control or Piezo1 siRNAs and stimulated with Piezo1 agonist Yoda1. Scale bar, 100 μm. (**C**) Time course of Yoda1-induced Ca^2+^ changes in control or Piezo1 siRNA-transfected human trabecular meshwork cells. (**D**) Yoda1-evoked intracellular Ca^2+^ elevation in human trabecular meshwork cells transfected with control or Piezo1 siRNAs. Data are expressed as the means ± SE (n = 4 experiments; ≥ 20 cells/experiment). **p < 0.01, Dunnett’s test.

To investigate the involvement of Piezo1 in response to cell stretch stimuli, primary hTM cells were transfected with Piezo1 or control siRNA. Piezo1 siRNA #1 or #2 transfection reduced Piezo1 mRNA levels in hTM cells to 42.1 ± 6.44% or 31.8 ± 4.31%, respectively, compared to the control (Fig. 2A). As Piezo1 is a Ca^2+^ permeable channel^11^, we evaluated Ca^2+^ influx induced by the Piezo1 agonist Yoda1 in hTM cells loaded with the Ca^2+^ indicator Fluo-8. The Yoda1-induced maximum fluorescence intensity change F_max_/F_0_ was 3.76 ± 0.07 in cells transfected with control siRNA, but 2.45 ± 0.05 or 2.20 ± 0.12 in cells transfected with Piezo1 siRNA # 1 or # 2, respectively (Fig. 2B–D). We confirmed that primary hTM cells expressed functional Piezo1 and that Piezo1 siRNA treatment significantly suppressed Piezo1 at the mRNA and functional levels.

### Mechanical stretch stimulation triggers calcium influx via Piezo1 activation in hTM cells

**Figure 3:**
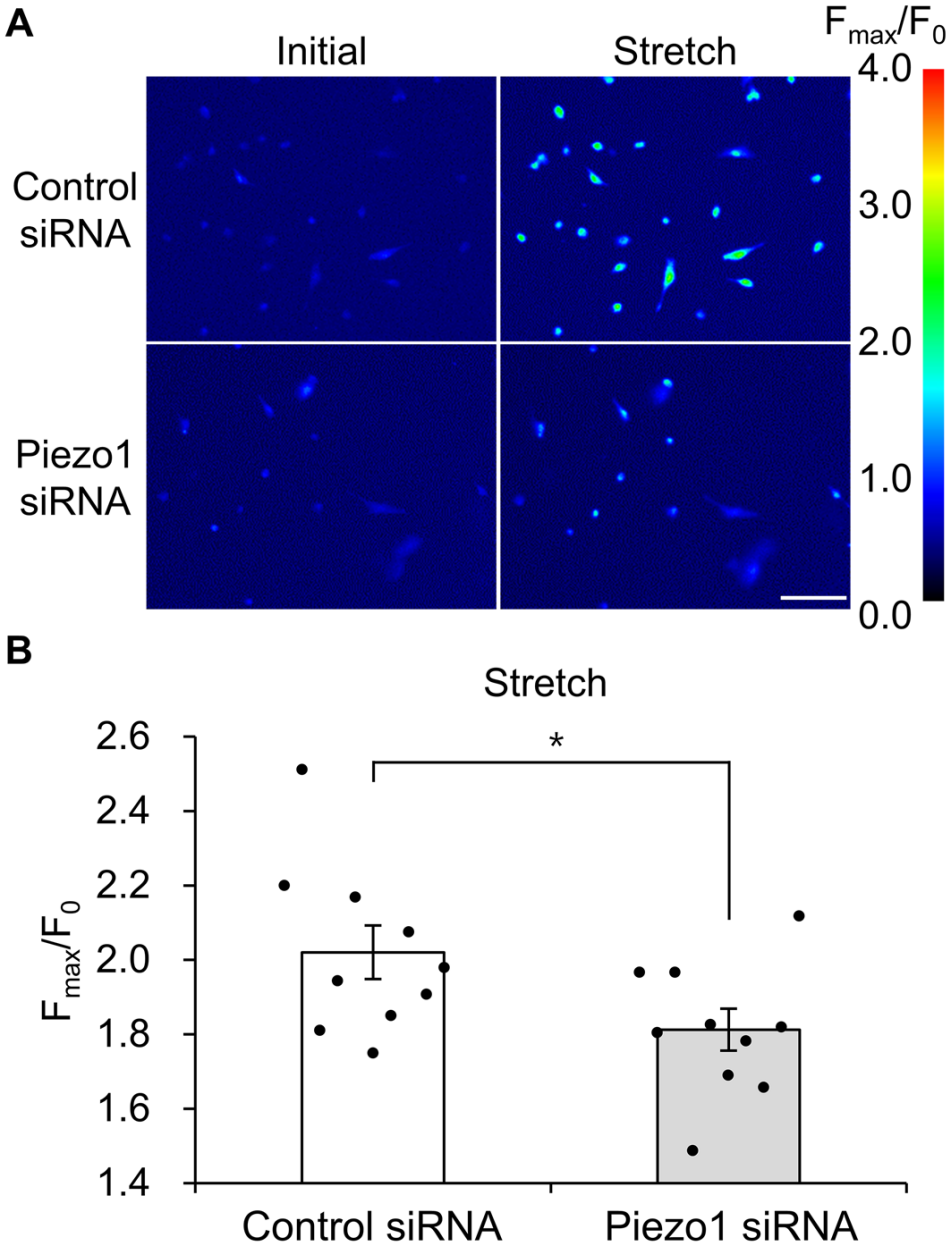
Intracellular Ca^2+^ changes upon stretch stimulation in human trabecular meshwork cells. (**A**) Images of intracellular Ca^2+^ changes upon stretch stimulation in human trabecular meshwork cells treated with control or Piezo1 siRNAs. Scale bar, 100 μm. (**B**) Intracellular Ca^2+^ levels following a single uniaxial stretch stimulation (1 way/s, 3.0 s pause) in human trabecular meshwork cells transfected with control or Piezo1 siRNA. Data are expressed as means ± SE (n = 5 experiments; ≥ 100 cells/experiment). *p < 0.05, two-tailed Student’s *t*-test.

Mechanical stretch stimulation in hTM cells promoted Ca^2+^ influx^10^. To investigate the relevance of Piezo1 in Ca^2+^ influx following mechanical stretch stimulation, we stimulated hTM cells grown on a fibronectin-coated silicon chamber with a 30% uniaxial stretch. Intracellular Ca^2+^ elevation induced by mechanical stretch was significantly suppressed in *PIEZO1*-knockdown cells (F_max_/F_0_ = 1.81 ± 5.65) compared with control cells (F_max_/F_0_ = 2.02 ± 7.19, Fig. 3A,B). These findings suggest that mechanical stretch stimulation in primary hTM cells induces Ca^2+^ influx in a Piezo1-dependent manner.

### Mechanical stretch stimulation in hTM cells promotes PGE_2_ release

**Figure 4:**
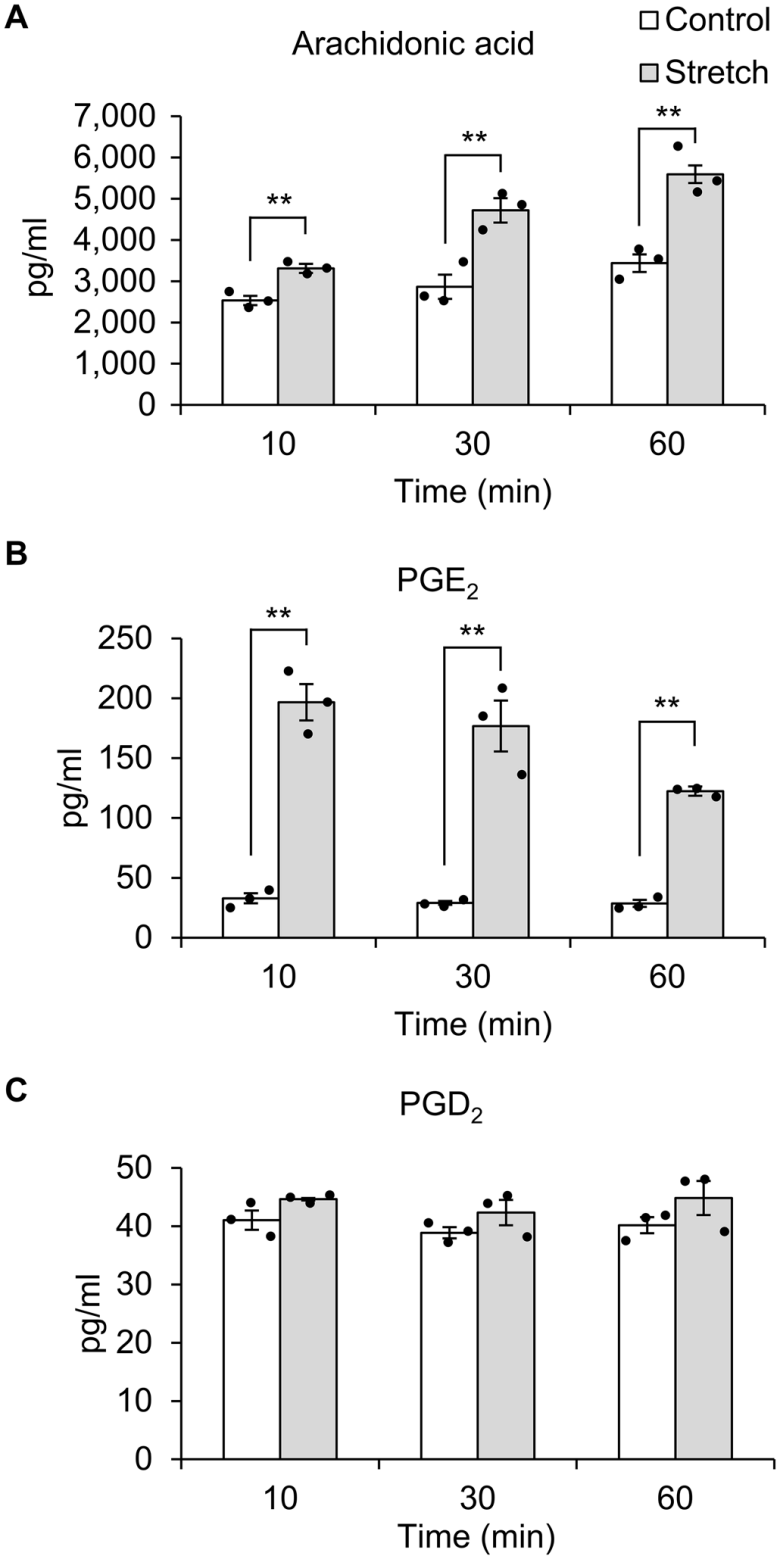
Mechanical stretch stimulation induces the release of arachidonic acid and PGE_2_ in human trabecular meshwork cells. Cell supernatants were collected 10, 30, and 60 min after a single uniaxial stretch stimulation and subjected to lipid analysis. Mechanical stretch stimulation in human trabecular meshwork cells triggered the release of arachidonic acid (**A**) and PGE_2_ (**B**), but not PGD_2_ (**C**). Data are expressed as means ± SE (n = 3 samples of lipid mediators extracted from cell supernatant in 3 chambers). **p < 0.01, two-tailed Student’s *t*-test.

**Figure 5:**
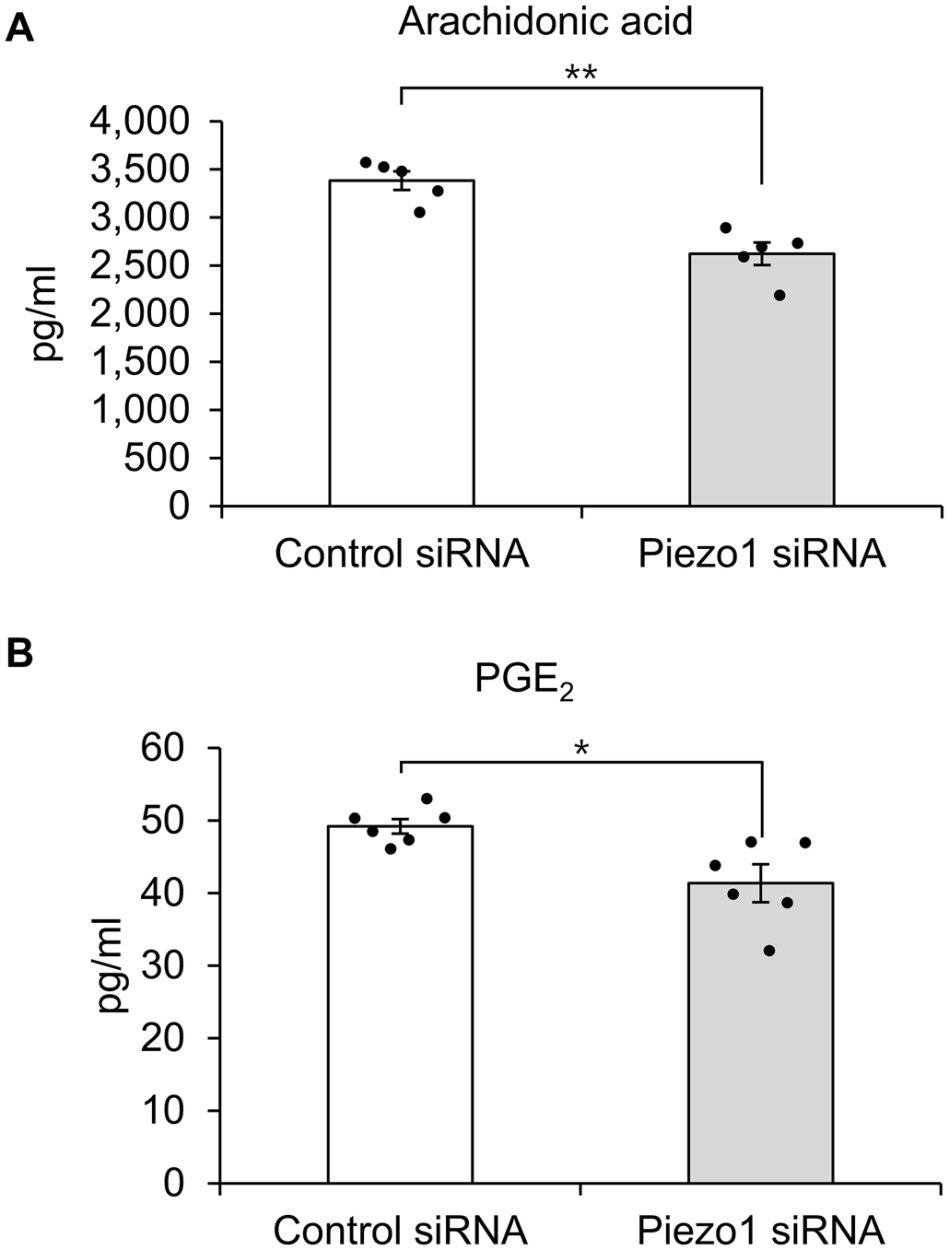
The impact of Piezo1 knockdown on the release of arachidonic acid and PGE_2_ following mechanical stretch stimulation in human trabecular meshwork cells. In human trabecular meshwork cells transfected with control or Piezo1 siRNAs, Cell supernatants were collected 10, 30, and 60 min after stretch stimulation. The levels of arachidonic acid (**A**), PGE_2_ (**B**) in the cell supernatant were measured. Piezo1 knockdown suppressed the release of arachidonic acid (**A**) and PGE_2_ (**B**). Data are expressed as mean ± SE (n = 3 samples of lipid mediators extracted from cell supernatant in 3 chambers). *p < 0.05. **p < 0.01, two-tailed Student’s *t*-test.

We then performed lipid analysis to investigate which lipid mediators are produced by hTM cells following mechanical stretch stimulation. We found that mechanical stretch stimulation significantly increased the amounts of arachidonic acid and PGE_2_ released from hTM cells (Fig. 4A,B). The amount of arachidonic acid in the cell supernatant after 10, 30, 60 min in the Control (No stretch) group was 2533.62 ± 111.49 pg/ml, 2864 ± 295.56 pg/ml, and 3437 ± 212.68 pg/ml, and in the Stretch group was 3309.48 ± 84.64 pg/ml, 4716.96 ± 258.83 pg/ml, and 5593.08 ± 331.87 pg/ml. The amount of PGE_2_ in the cell supernatant after 10, 30, 60 min in the Control (No stretch) group was 32.96 ± 4.24 pg/ml, 29.13 ± 1.60 pg/ml, and 28.69 ± 2.89 pg/ml, and in the Stretch group was 196.70 ± 15.15 pg/ml, 176.80 ± 21.27 pg/ml, and 122.40 ± 3.85 pg/ml. PGD_2_ release was not significantly altered by stretch (Fig. 4C), and other lipid mediators such as PGF_2α_ and TXB_2_ were not detected. Moreover, *PIEZO1* silencing in hTM cells significantly abrogated the mechanical stretch stimulation-induced release of arachidonic acid and PGE_2_ (Fig. 5A,B). The amount of lipid mediators in the supernatant of Conrol siRNA-treated cells by stretch stimulation was 3382.93 ± 96.34 pg/ml of arachidonic acid and 49.19 ± 1.10 pg/ml of PGE_2_, while that of Piezo1 siRNA-treated cells was 2622.05 ± 117.42 pg/ml of arachidonic acid and 41.35 ± 2.56 pg/ml of PGE_2_. These results suggesting that Piezo1 plays an essential role in the release of arachidonic acid and PGE_2_ following stretch stimulation.

### Yoda1 promotes PGE_2_ release in hTM cells

**Figure 6:**
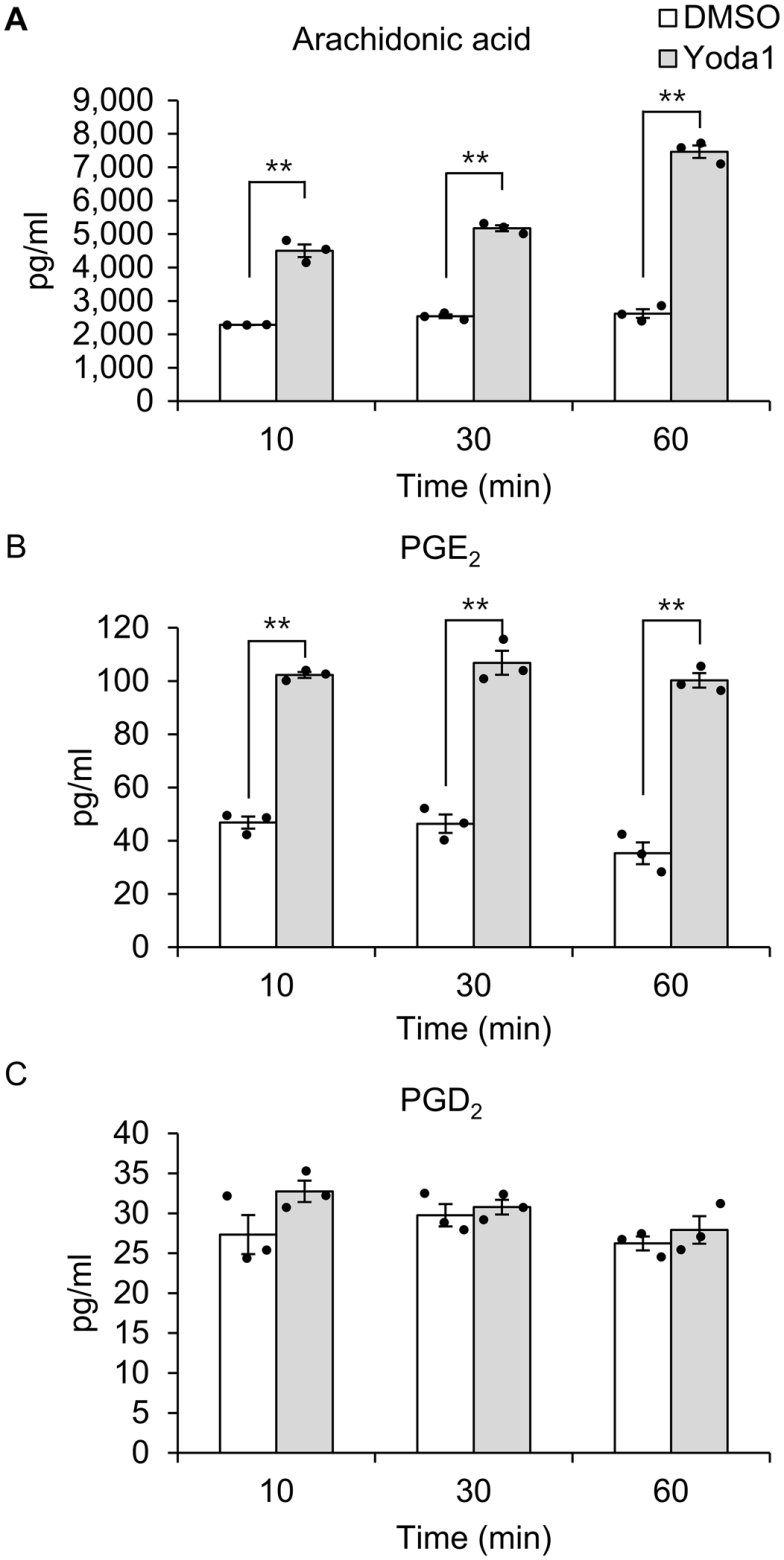
Direct activation of Piezo1 by Yoda1 induces the release of arachidonic acid and PGE_2_ in human trabecular meshwork cells. Human trabecular meshwork cells were incubated for the indicated time with or without Yoda1, and lipid mediators in the medium were analyzed. Yoda1 triggered the release of arachidonic acid (**A**) and PGE_2_ (**B**), but not PGD_2_ (**C**) in human trabecular meshwork cells. Data are expressed as the means ± SE (n = 3 samples of lipid mediators extracted from cell supernatant in 3 chambers). **p < 0.01, two-tailed Student’s *t*-test.

To assess the direct effect of Piezo1 activation, we measured lipid mediators in hTM cell supernatants after Yoda1 treatment. This analysis revealed that Yoda1 treatment significantly increased the levels of arachidonic acid and PGE_2_ released from hTM cells, but not PGD_2_ (Fig. 6). The amount of arachidonic acid in the cell supernatant after 10, 30, 60 min in the Control (DMSO) group was 2282.58 ± 3.20 pg/ml, 2539.86 ± 57.62 pg/ml, and 2620.76 ± 131.49 pg/ml, and in the Yoda1 group was 4500.13 ± 191.46 pg/ml, 5176.31 ± 89.15 pg/ml, and 7463.60 ± 188.21 pg/ml. The amount of PGE_2_ in the cell supernatant after 10, 30, 60 min in the Control (DMSO) group was 46.82 ± 2.27 pg/ml, 46.39 ± 3.43 pg/ml, and 35.28 ± 4.09 pg/ml, and in the Yoda1 group was 102.27 ± 1.12 pg/ml, 106.83 ± 4.52 pg/ml, and 100.25 ± 2.75 pg/ml. No other lipid mediators were detected. These data suggest that Piezo1 activation triggers the secretion of arachidonic acid and PGE_2_.

### Effects of Yoda1 and PGE_2_ on collagen gel contraction

**Figure 7:**
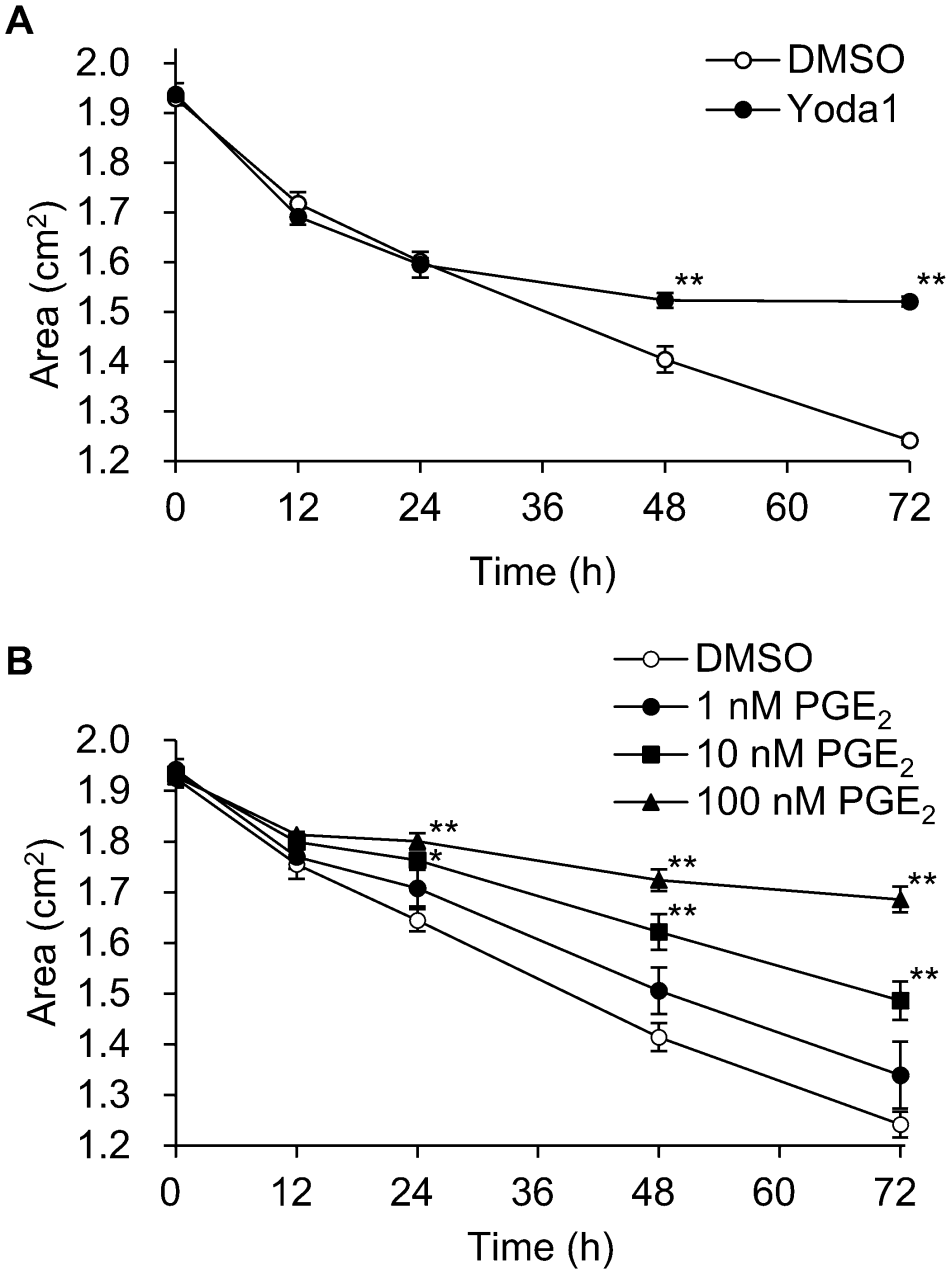
Effect of Yoda1 and PGE_2_ on human trabecular meshwork cell-mediated collagen gel contraction. Changes in the collagen gel area in presence of Yoda1 (**A**) and PGE_2_ (**B**). Data are expressed as means ± SE (n = 4 gels separated from 4 wells). *p < 0.05. **p < 0.01, two-tailed Student’s *t*-test or Dunnett’s test.

To investigate the effect of Yoda1 and the arachidonic acid metabolite PGE_2_ on hTM cells contraction, we performed an in vitro collagen gel contraction assay. This assay correlates that cells have contraction ability, which is supported by the fact that collagen alone does not cause contraction^21,22^. Compared to controls, Yoda1 significantly suppressed collagen gel contraction after 48 h and later, and PGE_2_ at 10 and 100 nM PGE_2_ after 24 h and later (Fig. 7A,B).

## Discussion

In this study, we demonstrated that Piezo1 was abundantly expressed in primary hTM cells, mediating mechanical stretch-induced Ca^2+^ influx and PGE_2_ release. Additionally, the Piezo1 agonist Yoda1 triggered Ca^2+^ influx and PGE_2_ release in hTM cells. Piezo1 activation in response to mechanical stimuli and subsequent PGE_2_ release suppressed hTM cell contraction. These results indicate that Piezo1 plays a crucial role in intracellular signaling induced by mechanical stretch and regulating TM contraction.

First, we analyzed the expression of *TRPV1-6* and *PIEZO1-2* in hTM cells and found that Piezo1 was most abundantly expressed at the mRNA level. Consistently, previous studies suggested that Piezo1 was one of 11 mechanotransduction channels identified in TM tissue and isolated TM cells^23^. Therefore, we hypothesized that Piezo1 might play a crucial role in TM physiology.

Next, we explored the role of Piezo1 signaling pathway in regulating TM contraction. As shown in Fig. 3, mechanical stretch-induced Ca^2+^ influx was inhibited when *PIEZO1* was silenced. This finding is consistent with previous findings in urothelial cells^12^, red blood cell^13^, and neural stem cells^14^, suggesting that Piezo1 constitutes Ca^2+^ influx pathway in mechanical stretch of hTM cells. Arachidonic acid and its metabolites have been reported to play important roles in mechanical signaling^15,16^. Additionally, cyclic mechanical stress induced the release of arachidonic acid and its metabolic product PGE_2_ in porcine TM cells^24^. Therefore, we next investigated the profile of lipid mediators released after mechanical stretch and found a significant increase in the levels of arachidonic acid and PGE_2_ secreted from primary hTM cells following mechanical stretch (Fig. 4). We further found that the stretch-induced secretion of arachidonic acid and PGE_2_ was abrogated by *PIEZO1* silencing (Fig. 5), and pharmacological activation of Piezo1 with Yoda1 triggered the release of arachidonic acid and PGE_2_ in hTM (Fig. 6), suggesting an essential role of Piezo1 in the mechanical stretch-induced secretion of these lipid mediators.

**Figure 8:**
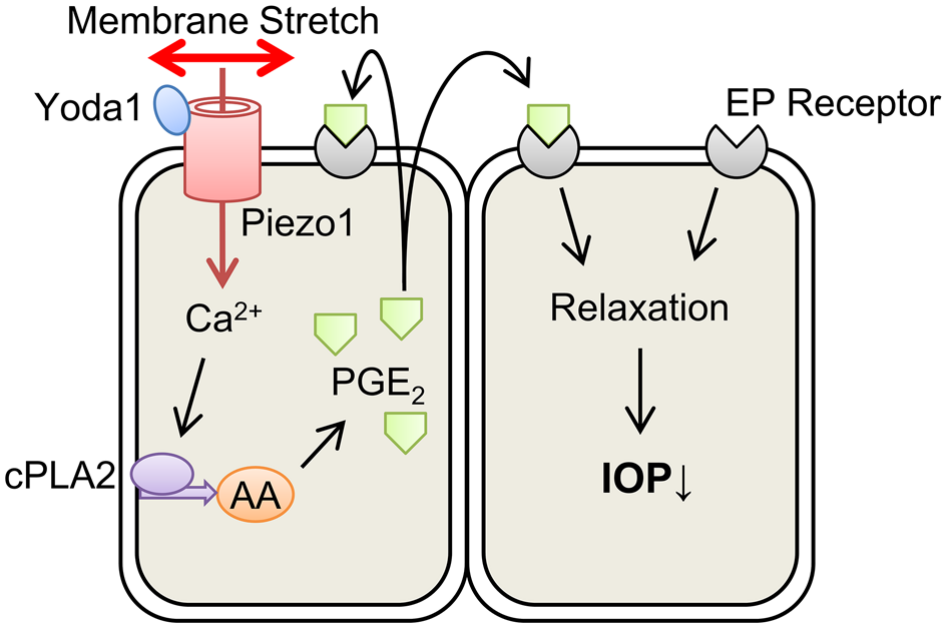
Proposed model of how Piezo1 activation contributes IOP regulation in human trabecular meshwork cells. Membrane stretch activates Piezo1 on hTM cells, causing Ca^2+^ influx. This increase in intracellular Ca^2+^ activates cytosolic phospholipase A2 (cPLA2) and releases arachidonic acid (AA) from membrane phospholipids. The liberated arachidonic acid is metabolized to produce PGE_2_, which causes cell relaxation via EP receptor, leading to a decrease in IOP.

Moreover, we found that PGE_2_ produced by Piezo1 activation inhibited hTM contraction. The contraction of TM cells decreases the size of intercellular spaces and permeability, resulting in reduces aqueous humor outflow^25^. Agents that induce contraction of TM cells have been shown to increase IOP by reducing the outflow rate of aqueous humor, whereas relaxation of TM cells increases outflow and lowers IOP^26–30^. Consistent with these, EP2 agonists inhibited gel contraction^29^ and have an IOP lowering effect in vivo^18,30,31^. The response of TM cells by mechanical stimulation leading to the release of PGE_2_ may corroborate that recently introduced EP2 agonist, omidenepag, and previously reported EP2 agonists can significantly reduce IOP^30,31^. Our result suggests new homeostatic mechanism in the eye that elevated IOP stretches TM and subsequently TM cells respond to protect eyes through enhancing aqueous outflow via endogenously produced PGE_2_. The proposed mechanism underlying Stretch-mediated Piezo1 activation in human trabecular meshwork cells is diagrammatically summarized in Fig. 8.

To assess the effect of direct activation of Piezo1 in hTM cells, we used the Piezo1-selective activator Yoda1. Yoda1 was identified among approximately 3.25 million compounds as a synthetic small molecule that acts as an agonist of both human and mouse Piezo1^32^. We investigated the effect of Yoda1 on Piezo1 activation in hTM cells and confirmed that it can be reliably activated at 10 μM (Fig. 2). Previous studies showed that high concentration (10–100 μM) of Yoda1 simultaneously caused substantial cytotoxicity and compromise of the HUVEC monolayer after 24 h^33^. In this study, we found that 10 μM Yoda1 reduced survival by approximately 10% after 24 h (See Supplementary Fig. S1 online). Although 10 μM Yoda1 significantly inhibited hTM cell contraction after 48–72 h in the gel contraction assay (Fig. 7A), we also showed the effect of PGE_2_ in gel contraction assay because of prolonged 10 μM Yoda1exposure (72 h) not only activates Piezo1 but may also have toxic effects. As shown in Fig. 7B, PGE_2_ significantly inhibited hTM collagen gel contraction in a dose-dependent manner, suggesting PGE_2_ production after Piezo1 activation contributes to IOP regulation. On the other hand, activation of EP_2_ (and EP_1_, EP_4_) receptors have been demonstrated to increase TM cell contractility^34^. However, this result is a response within 1 h of agonist exposure. PGE_2_ lowers IOP, and also temporarily increases IOP in the early stages after instillation^18^. The increase in TM cell contractility due to activation of EP_2_ receptor may reflect this transient increase in IOP and change at later time. EP_2_ receptor was also expressed in Schlemm's canal (SC) and its activation was shown to reduce SC cell stiffness^34^. Accordingly, PGE_2_ released from hTM cells may target SC in addition to TM.

While our findings highlighted the critical role of Piezo1 in IOP regulations upon TM stretch, there were several limitations in the present study. Piezo1 and TRPV4 were compared only for mRNA expression in this study. There are several reports comparing the role of Piezo1 and TRPV4 in response to stretch stimulation. In bladder urthelium, the sensitivity of Piezo1 to stretch stimuli was higher than that of TRPV4^12^. In chondrocyte, Piezo1 was largely responsible for the stretch-activated current, whilst TRPV4 had no involvement in the specific mechanoelectrical transduction pathway^35^. However, there are no reports comparing the importance of Piezo1 and TRPV4 in TM. Thus, future studies are required to compare the sensitivity of Piezo1 and TRPV4 to stretch stimuli, as well as the relationship between Piezo1 and TRPV4 mRNA levels and function. We focused on lipid mediator and its involvement in the TM contraction, and didn’t further explore whether Piezo1 are involved in the stretch-induced cytoskeletal remodeling in hTM cells, which should be determined in the future study. In addition to lipid mediators, nitric oxide (NO) is a candidate that link intracellular Ca^2+^ elevation and regulation of TM outflow. NO is formed by the enzyme called nitric oxide synthase (NOS) and Three different isoforms have been identified including neuronal NOS or brain NOS (nNOS, bNOS or NOS1), endothelial NOS (eNOS or NOS3), and inducible NOS (iNOS or NOS2). nNOS1 and eNOS are constitutively expressed in cells and are activated by an increase in intracellular Ca^2+^^36^. iNOS is induced by a variety of stimuli, such as endotoxins and inflammatory cytokines, regardless of Ca^2+^ level. Three isoforms of NOS has been reported to be expressed in TM^37^. Several studies in vitro and in vivo have demonstrated IOP-lowering effects of NO, and treatment with the ROCK inhibitor Y-26732 increased the expression of eNOS and NO production more significantly^38,39^. Increased visual field damage results in decreased eNOS expression and Ca^2+^-dependent NOS activity in TM of POAG patients^40^. Thus, elevated intracellular Ca^2+^ in TM may increase eNOS expression and NO production, leading to decreased IOP. In this study, we applied a uniaxial stretch stimuli to Piezo1 knockdown cells, but by applying a multiaxial stretch stimuli to knockout cells, the exact contribution of Piezo1 in the cellular response to stretch stimuli could be obtained under more physiological conditions. Moreover, since our conclusions are based solely on evidence from in vitro experiments, we couldn’t determine the pathophysiological relevance of our in vitro observations in regulation of IOP or outflow facility. Further study using animal models or perfusion experiment will be needed to determine this issue.

In conclusion, this study clarified the role of new aqueous humor outflow regulation mediated by Piezo1 in TM. The present data has shown that Piezo1 regulates Ca^2+^ influx in response to mechanical stretching and that PGE_2_ is essential for Piezo1-mediated hTM cell contraction inhibition. Our study revealed a novel role for Piezo1 in hTM cells and proposed a new therapeutic strategy for the conventional outflow pathway.

## Methods

### Cell culture

Primary human trabecular meshwork (hTM) cells were isolated from human corneal scleral rims obtained from the Rocky Mountain Lions Eye Bank from 8 donors (both male and female, the mean age ± SD was 47.3 ± 25.9), as described previously^41^. All experiments were conducted according to the principles of the Declaration of Helsinki and approved by the ethics committee of the University of Tokyo. Written informed consent was obtained from donors. Briefly, corneal scleral rims maintained in Optisol were cut and divided into 6 to 8 sections. The trabecular meshwork strip was picked and isolated with forceps from sections. TM strips were cultured in a dish coated with collagen gel (Cellmatrix Type I-A, Nitta Gelatin Inc., Osaka, Japan). After the TM cells migrated from the tissue, the gel was digested with collagenase, and the collected TM cells were grown on dishes coated with 1 μg/mL fibronectin. hTM cells from passages 3 to 6 were used in subsequent experiments and maintained in Trabecular Meshwork Cell Medium (ScienCell Research Laboratories, Carlsbad, CA) ) containing 2% fetal bovine serum (ScienCell), 1% Trabecular Meshwork cell growth supplement (ScienCell) and 1% penicillin/streptomycin solution (ScienCell). The identity of the isolated cells was confirmed by the dexamethasone-induced upregulation of *MYOC*. After 7 days of exposure to vehicle (DMSO), 100 or 500 nM dexamethasone, dexamethasone enhanced *MYOC* expression (see Supplementary Fig. S2 online). Cells were transfected with *PIEZO1*-targeting siRNAs (SASI_Hs01_00208584, SASI_Hs01_00208585, Merck, Darmstadt, Germany) or MISSION siRNA Universal Negative Control #1 siRNAs (Merck). siRNA transfections were performed using MISSION siRNA Transfection Reagent (Merck) according to the manufacturer’s instructions.

### Quantitative real-time polymerase chain reaction (qPCR) analysis

Total RNA was extracted from hTM cells at 24 h after siRNA transfection using ISOGEN (Nippon Gene, Tokyo, Japan). The concentration of Total RNA was measured by the absorbance at a wavelength of 260 nm using a spectrophotometer NanoDrop 2000 (Thermofisher, Waltham, MA). Complementary DNA (cDNA) was prepared using ReverTra Ace qPCR RT Master Mix with gDNA remover (Toyobo, Osaka, Japan). Quantitative real-time PCR was run on a Thermal Cycler Dice Realtime System (Takara Bio Inc., Shiga, Japan) using SYBR Premix Ex Taq™ II (Tli RNaseH Plus) (Takara Bio Inc.). Standard curves were used to determine mRNA transcript copy number in individual reactions. Primers specific for *TRPV1-6* and *PIEZO1-2* were designed by us or purchased from Takara Bio Inc., as detailed in Table 1.

**Table 1:** Primers specific for *TRPV1-6*, *PIEZO1-2*, *GAPDH*, and *MYOC*.

Gene Name	Primer	Sequences (5′–3′)	Product Size(bp)
*TRPV1*	Forward	AAGTTCCTGCTGCAGAACTC	130
	Reverse	TGCTCGTCACAAACTTCGTG	
*TRPV2*	Forward	AAACTGCTGCAGGCGAAATG	95
	Reverse	GCAACAGCGGTGAAGATGAAC	
*TRPV3*	Forward	GTTAGCTACCCGCATTAAGCCTGA	107
	Reverse	AGCAATTCTGGAATTCCCAGCTC	
*TRPV4*	Forward	TGCATGCGCCACCATTTTTG	114
	Reverse	TATTGAGCACCGGCAAATCC	
*TRPV5*	Forward	GGAGACCTAATGCGTTTCTGCTG	197
	Reverse	AAGGGCAAGTCCACGTCGTA	
*TRPV6*	Forward	AGCAGTGCCAATTGGGAAAG	121
	Reverse	TGAGAACACGCAGTCAGATCTG	
*PIEZO1*	Forward	ATGCCAACGAGAAGCACATG	128
	Reverse	ACGGATGTACTTGGGGAAGAG	
*PIEZO2*	Forward	ATTTCATTGTGCGGCCCAAC	127
	Reverse	TTGTCACCTGCCATGATTCG	
*GAPDH*	Forward	AATTCCATGGCACCGTCAAG	104
	Reverse	ATCGCCCCACTTGATTTTGG	
*MYOC*	Forward	TACACGGACATTGACTTGGC	159
	Reverse	ATTGGCGACTGACTGCTTAC	

### Calcium imaging and mechanical cell stretch stimulation

hTM cells or those 24 h after siRNA transfection were seeded on cover glass chambers (Iwaki, Shizuoka, Japan) or stretch chamber STB-CH-24 (STREX Inc., Osaka, Japan) coated with 1 μg/mL fibronectin. After washing with PBS, hTM were loaded with 0.1% BSA (Sigma) and 5 μM fluorescent Ca^2+^ indicator Fluo-8 AM (AAT Bioquest, Inc., Sunnyvale, CA) for 20 min. Subsequently, 10 μM Yoda1 (Cayman Chemical, Ann Arbor, MI)^32^ was applied directly to the cover glass chambers. STB-CH-24 was attached to a stretch device STB-150 (STB-150 in this study was modified to stretch up to 40% using STB-CH-24; STREX Inc.) on the stage of a fluorescence microscope and subjected to a single uniaxial stretch with a nominal 30% extension (1 way/s, 3.0 s pause) at room temperature. As evaluated from images of the chamber at 30% extension, the actual extension of the cell culture surface was about 20%. Fluorescent images were acquired under a fluorescence microscope (Keyence, Osaka, Japan). The fluorescence intensity of Fluo-8 was quantified using ImageJ software (https://imagej.nih.gov/ij/download.html). First, the captured images were stacked and the misalignment was corrected with the StackReg plugin. Next, the image with the highest fluorescence intensity from the cells was binarized by setting a threshold value by the triangle method, and the cells were selected with Analyze particles. Finally, the fluorescence intensity of the selected cells was measured on all images. The change ratio (F_T_/F_0_, F_max_/F_0_) was calculated using peak and basal values. F_T_, F_max_, and F_0_ represent the fluorescence intensity at that time, the maximum fluorescence intensity, and the fluorescence intensity before stimulation, respectively.

### Lipid analysis

Lipid analysis was performed as previously described^42^. Cell supernatant was collected 10, 30, and 60 min after 10 μM Yoda1 treatment or stretch stimulation, and stored at − 80 ℃. Methanol was added to each sample at a ratio of 1:1, and 10 μL of internal standard was added. The internal standard contained 6-keto-PGF_1α_-d4, AEA-d4, arachidonic acid-d8, docosahexaenoic acid-d5, eicosapentaenoic acid-d5, OEA-d4, tetranor-PGEM-d6, TxB_2_-d4, PGF_2α_-d9, PGE_2_-d4, PGD_2_-d4, LTB_4_-d4, LTC_4_-d5, LTD_4_-d5, LTE_4_-d5, 5(S)-HETE-d8, 12-HETE-d8, 15(S)-HETE-d8, and PAF-C16-d4 (Cayman Chemical). After vortexing, samples were centrifuged at 15,000×*g* and 4 °C for 15 min to obtain phase separation. Supernatants were transferred into 15 mL glass tubes. The supernatant and 0.03% formic acid were mixed at a ratio of 1:4 with vortex. Samples were loaded onto a solid-phase extraction column (Oasis HLB, Waters Corporation, Milford, MA) pre-conditioned with methanol and 0.03% formic acid. The column was centrifuged at 800 rpm and 4 °C for 2 min, and washed with petroleum ether. Methanol containing 0.2% formic acid was added to the column, followed by centrifugation at 800 rpm and 4 °C for 2 min to elute lipids. After evaporation, eluents were reconstituted in methanol.

The samples were analyzed on a Nexera ultra-high performance liquid chromatograph connected to a triple quadrupole mass spectrometer LCMS-8060 (Shimazu, Kyoto, Japan). Chromatographic separation was performed using a Kinetex C8 column (2.6 μm, 2.1 × 150 mm, Phenomenex, Torrance, CA). The mass spectrometer was operated in selected reaction monitoring mode allowing for simultaneous detection of target lipid mediators. Quantification was performed by internal standard calibration method using chromatographic peak areas, as previously described^42^.

### Gel contraction assay

Gel contraction assay was performed using the Collagen Gel Culturing Kit (Nitta Gelatin, Inc., Osaka, Japan) as per the manufacturer’s instructions. Collagen type I, 10 × MEM, and reconstitution buffer (pH 7.3) were mixed at a ratio of 7:1:1 at 4 °C. The final hTM cell concentration was adjusted to 1 × 10^6^ cells/mL. The resultant mixture and cell suspension were mixed at a 9:1 ratio and 500 μL of the mixture was added to each well of 24-well plates and incubated at 37 °C to allow for collagen gel formation. After 60 min, gels were placed in 6-cm dishes containing 5 mL Trabecular Meshwork Cell Medium with DMSO, 10 μM Yoda1, or PGE_2_ (1, 10, or 100 nM). After 0, 12, 24, 48, and 72 h, gels were imaged with a gel imaging device, and the area was quantified with ImageJ software (https://imagej.nih.gov/ij/download.html).

### Statistical analysis

Data were represented as mean ± standard deviation (SD) in Fig. 1, or as mean ± standard error (SE) in Figs. 2, 3, 4, 5, 6, 7 and Supplementary Figure with individual data plotted. Statistical analyses were performed using a two-tailed Student’s *t*-test or Dunnett’s test. A difference was considered statistically significant when *p* < 0.05.

## Supplementary Information

Supplementary Figure Legends.

Supplementary Figure S1.

Supplementary Figure S2.
