# Supplementary material for: Mechanical stretch induces Ca2+ influx and extracellular release of PGE2 through Piezo1 activation in trabecular meshwork cells
Source: Sci Rep. 2021 Feb 17;11:4044. doi: 10.1038/s41598-021-83713-z (PMC7890064; doi:10.1038/s41598-021-83713-z)
Supplement: Supplementary file 1 — Supplementary Figure Legends. [file 41598_2021_83713_MOESM1_ESM.docx]

**Supplementary Information**

**Mechanical stretch induces Ca^2+^ influx and extracellular release of PGE_2_ through Piezo1 activation in Trabecular Meshwork Cells**

Takatoshi Uchida^1, 2^, Shota Shimizu^1, 2^, Reiko Yamagishi^1^, Suzumi M. Tokuoka^3^, Yoshihiro Kita^3, 4^, Megumi Honjo^1^, Makoto Aihara^1, *^

^1^Department of Ophthalmology, Graduate School of Medicine, the University of Tokyo, Tokyo, Japan

^2^Senju Laboratory of Ocular Science, Senju Pharmaceutical Co., Ltd., Kobe, Japan

^3^Department of Lipidomics, Graduate School of Medicine, the University of Tokyo, Tokyo, Japan

^4^Life Science Core Facility, Graduate School of Medicine, the University of Tokyo, Tokyo, Japan

**Supplementary Figure Legends**

**Supplementary Figure S1. The effect of Yoda1 on proliferation of** **primary human trabecular meshwork cells.**

Primary human trabecular meshwork cells were treated with vehicle (DMSO) or Yoda1 for 24 hours, and proliferation was evaluated by WST-8 assay. Data are presented as means ± SE (n = 4). ^*^*p* < 0.05. ^**^*p* < 0.01, Dunnett’s test.

**Supplementary Figure S2. Dexamethasone induced MYOC expression in primary human trabecular meshwork cells.**

Primary human trabecular meshwork cells were treated with vehicle (DMSO), 100 or 500 nM dexamethasone for 7days and mRNA levels were determined by qPCR. Data are presented as means ± SE (n = 4). ^*^*p* < 0.05. ^**^*p* < 0.01, Dunnett’s test.
