# Supplementary figures and images for: Mechanical stretch induces Ca2+ influx and extracellular release of PGE2 through Piezo1 activation in trabecular meshwork cells
Source: Sci Rep. 2021 Feb 17;11:4044. doi: 10.1038/s41598-021-83713-z (PMC7890064; doi:10.1038/s41598-021-83713-z)

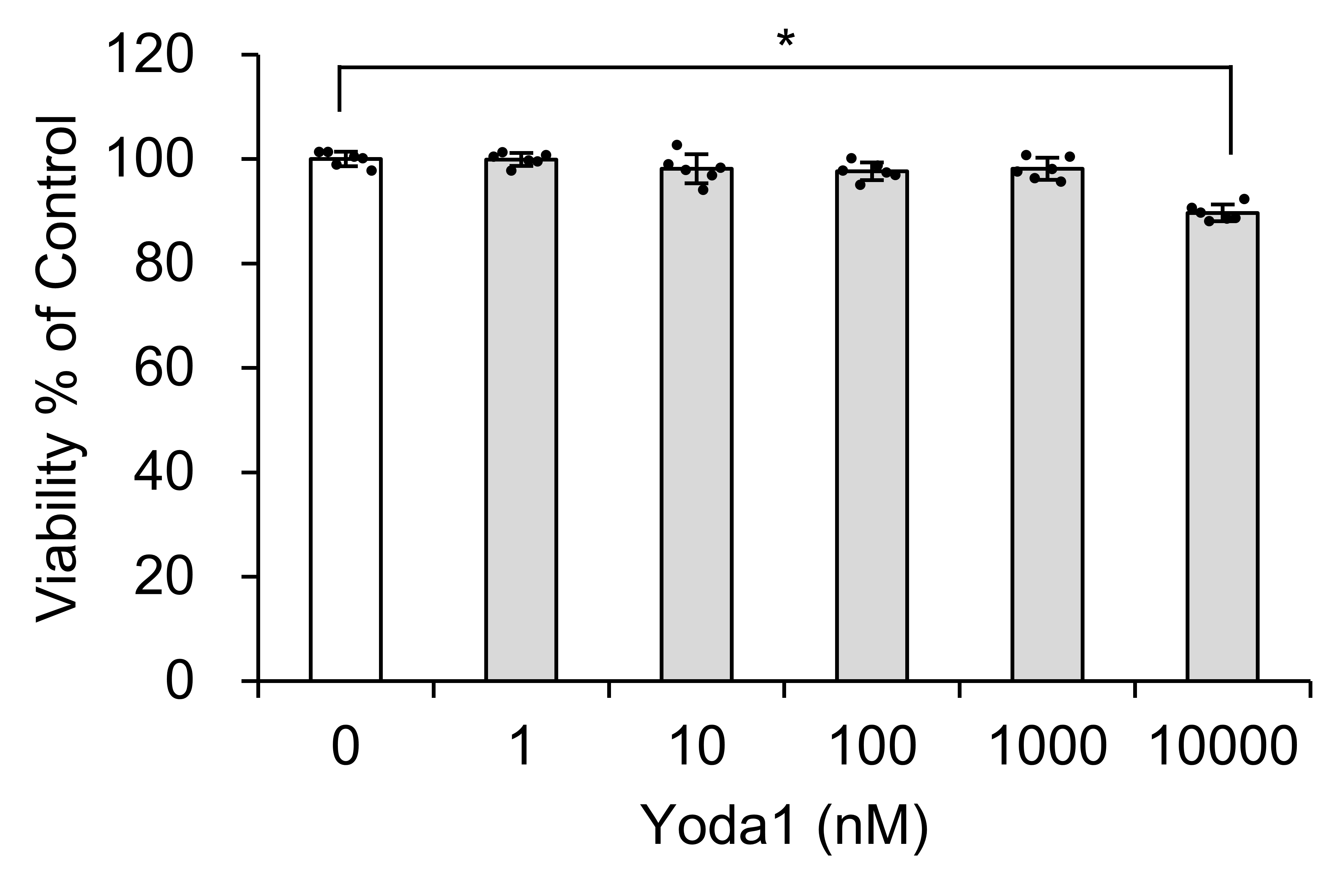

Supplement: Supplementary file 2 — Supplementary Figure S1. [file 41598_2021_83713_MOESM2_ESM.tif]

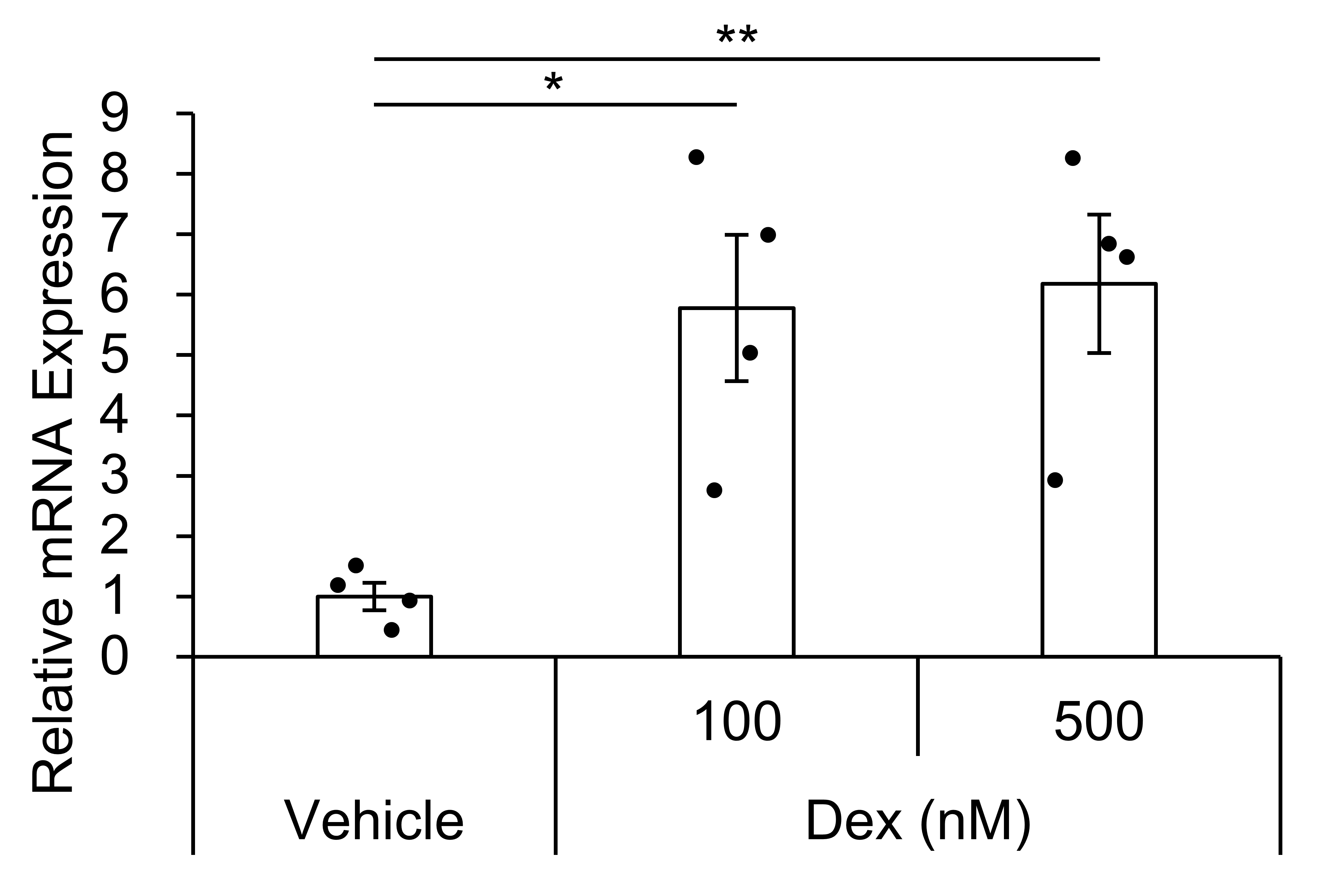

Supplement: Supplementary file 3 — Supplementary Figure S2. [file 41598_2021_83713_MOESM3_ESM.tif]
